# Immunomodulatory effects of a new whole ingredients extract from Astragalus: a combined evaluation on chemistry and pharmacology

**DOI:** 10.1186/s13020-019-0234-0

**Published:** 2019-03-27

**Authors:** Zhi Xin Li, Guan Ding Zhao, Wei Xiong, Ke Gang Linghu, Qiu Shuo Ma, Wai San Cheang, Hua Yu, Yitao Wang

**Affiliations:** 1 Institute of Chinese Medical Sciences, State Key Laboratory of Quality Research in Chinese Medicine, University of Macau, Macao, China; 2 HKBU Shenzhen Research Center, Shenzhen, Guangdong China; 3 School of Chinese Medicine, Hong Kong Baptist University, Kowloon Tong, Hong Kong China; 4 Institute of Chinese Medical Sciences, University of Macau, Room 8008, Building N22, Avenida da Universidade, Taipa, Macao SAR China; 5 Institute of Chinese Medical Sciences, University of Macau, Room 1050, Building N22, Avenida da Universidade, Taipa, Macao SAR China

**Keywords:** Whole ingredients extract, Chemical integrity, Immunomodulation, Astragali Radix

## Abstract

**Background:**

Water extract (WAE) and ultrafine powder (UFP) are two types of commonly used supplements in preparing various pharmaceutical products and functional foods. However, the correlations of the chemical compositions with the traditional functions between WAE and the herb itself, as well as the potential problems of safety for UFP have been more and more concerned by many doctors and customers.

**Methods:**

In this study, a new whole ingredients extract of Astragalus (WIE) was prepared using the gradient solvent extraction method. The chemical compositions of WIE and WAE were comparatively analysed using spectrophotometric and chromatographic approaches. In addition, the in vivo immunomodulatory effect of WIE, WAE and UFP of Astragalus were comprehensively compared in cyclophosphamide (Cy)-induced immunosuppressive mice.

**Results:**

The compositions and contents of main active ingredients (polysaccharides, saponins and flavonoids) in WIE were determined to be more abundant than those in WAE. In Cy-induced immunosuppressive mice, oral administered with low dosage of WIE (equalled to 1.0 g herb/kg/day) for 18 consecutive days significantly improved the immune-related responses (body weight, number of peripheral white blood cells, thymus and spleen indexes, splenocyte proliferations, natural killer cell activity, splenic lymphocyte subset, and serum levels of immunoglobulins G and M). The potency of three Astragalus preparations on immunomodulation was observed to be WIE ≥ UFP > WAE.

**Conclusions:**

WIE maximally retained the chemical integrity of astragalus, and presented better therapeutic effectiveness than UFP and WAE. It can be further developed as new strategy for reasonable use of medicinal/edible herb-derived supplement (extract) for pharmaceutical and healthcare applications.

**Electronic supplementary material:**

The online version of this article (10.1186/s13020-019-0234-0) contains supplementary material, which is available to authorized users.

## Background

Immune system plays an important role affecting on the health and life quality of people. Nature-sourced products with functions of immunomodulation are more and more favourite by many people for the purposes of healthcare. Although numerous of herbal products have been developed and commercialized currently with various or specific functions, their effects on healthcare are highly depended on their preparation processing, especially the methods for herbal extraction.

Traditionally, prolonged boiling or ‘decocting’ using water as solvent is the earliest and most popular method for preparing herbal extracts [1]. The water extract (WAE) are commonly used as the raw materials or supplements in preparing various pharmaceutical and healthcare products. Since the physicochemical properties of the ingredients in herbal medicines are significantly different, such as the water-soluble ingredients (polar macromolecules and small molecules) and alcohol-soluble ingredients (weak polar and non-polar small molecules), the contents of alcohol-soluble ingredients in water extract are very limited. Therefore, compare to the herb itself, WAE contains incomplete chemical ingredients thus resulting the loss of integrity in corresponding pharmacological activities.

In recent years, ultrafine powder (UFP) of herbal materials prepared using ultrafine grinding technology has been quickly developed [2]. The UFP contains herbal particles in small diameter (less than 45 μm, up to 90% of the sample) which are mostly broken in cytoderm level. The ultrafine grinding provides UFP nice surface properties such as the higher dispersibility and specific surface area [3], thus presenting better therapeutic efficacy of the herbs [4]. Moreover, UFP retains higher integrity in chemical composition keeping both water-soluble and alcohol-soluble ingredients [5] and improves their dissolution and absorption in gastrointestinal tract [6, 7]. However, the safety problems of UFP have raised attention [8]. The small particle size of UFP may adhere to the gastrointestinal mucosa, affecting gastrointestinal peristalsis, mucosal absorption, and secretion of gastrointestinal hormones. After the cell wall-broken, some new side effects may occur because the dissolution of the active ingredient as well as some other unexpected components [9]. Air, moisture and charge are more likely to be absorbed onto the powder surface and thereby form an unstable state [10]. In addition, the large amount in therapeutic doses of UFP restrict the compliance of their clinical use [1].

The aforementioned approaches to prepare raw material or food supplement for pharmaceutical products and functional foods have different disadvantages which need to be further improved. Therefore, developing more effective herbal extracts with good integrity in active ingredients as well as good safety and satisfied compliance is of high importance for enhancing the quality and health-protective effects of herbal products. In the present study, a new whole ingredients extract (WIE) system with Astragalus as a case study was prepared using gradient solvent extraction method. The chemical compositions of WIE and WAE were comparatively analysed using spectrophotometric and chromatographic approaches. Moreover, the in vivo immunomodulatory effects of WIE, WAE and UFP of Astragalus were comprehensively compared in cyclophosphamide (Cy)-induced immunosuppressive mice.

## Methods

The Minimum Standards of Reporting Checklist contains details of the experimental design, and statistics, and resources used in this study (Additional file 1).

### Chemicals and reagents

Astragaloside IV, calycosin-7-O-β-d-glucoside, ononin, calycosin and formononetin (the purities of all standards were higher than 98% by HPLC analysis) were purchased from Chengdu Pufeide Biotech Co., Ltd. (Chengdu, China). Acetonitrile (ACN) as HPLC grade was purchased from Merck (Darmstadt, Germany). Cyclophosphamide (Cy), trypan blue, lipopolysaccharide (LPS) and concanavalin A (ConA) were purchased from Sigma-Aldrich Co. (St. Louis, MO, USA). Milli-Q water was prepared using a Milli-Q system (Millipore, MA, USA). All other chemicals used were analytical grade and purchased from Sigma-Aldrich (St. Louis, MO, USA).

Enhanced Cell Counting Kit-8 (CCK-8), ACK Lysis Buffer and 1% Nonidet P-40 (NP-40) were obtained from the Beyotime Institute of Biotechnology (Jiangsu, China). Immunoglobulin G (IgG), and immunoglobulin M (IgM) kits were all obtained from Nanjing Jiancheng Bioengineering Institute (Nanjing, China). Rat anti-mouse CD3-FITC, anti-mouse CD4-APC, and anti-mouse CD8a-PerCP-Cy5.5 were provided by eBioscience (San Diego, CA, USA). All materials for cell culture were obtained from Thermo Fisher Scientific (Waltham, MA, USA).

### Herbs and herbal extracts

Astragali Radix (Astragalus, *Astragalus membranaceus* (Fisch.) Bge. var. *mongholicus* (Bge.) Hsiao) and its ultrafine powder (UFP) with D90 < 45 μm were both purchased from Guangjitang CSPC Pharm Group (Guizhou, China). The herb sample was authenticated by the corresponding author and the voucher specimen (HQ-2017001) was deposited in Institute of Chinese Medical Sciences, University of Macau.

For WIE, the air-dried and powdered Astragalus (400 g) was gradient extracted with 95% ethanol (4 L), 50% ethanol (4 L) and water (4 L) at 60 °C for 1 h for each. The filtered extracts were combined and concentrated under rotate reduced pressure to remove ethanol. The concentrated extract was then lyophilized with a Virtis Freeze Dryer (The Virtis Company, New York, USA). The powder of WIE (yield: 31.27%) was kept at 4 °C for further experiments.

For WAE, the air-dried and powdered Astragalus (400 g) was extracted thrice with water (4 L) at 60 °C for 1 h for each. The combined extract was filtered, concentrated and then lyophilized with a Virtis Freeze Dryer. The powder of WAE (yield: 30.43%) was kept at 4 °C for further experiments.

### Characterization of the extracts

#### Total polysaccharides

The contents of total polysaccharides in WIE and WAE were determined using phenol–sulfuric acid method. Briefly, a 2 mL of glucose solution (0–50 μg/mL) or sample solution (1 mg/mL) was mixed with 1 mL of 6% phenol solution, and then incubated at 60 °C for another 15 min after addition of 5 mL concentrated sulfuric acid. After cooling, the absorbance was measured at 490 nm. The content of total polysaccharides in WIE and WAE were calculated using glucose as standard.

#### Total saponins and Astragaloside IV

The total saponins in WIE and WAE were determined using Vanillin (glacial acetic acid) assay. Briefly, 1 mL of WIE or WAE solution (1 mg/mL in water) was loaded onto an activated SepPak C18 Cartridges (Waters Corp., Milford, USA) and then washed with 2 mL of water. The adsorbed saponins were eluted with 1 mL methanol into a glass tube. After evaporation, the residue was dissolved in 0.2 mL 5% vanillin in glacial acetic acid solution and 0.8 mL perchloric acid. Subsequently, the mixture was incubated at 60 °C for 15 min followed by addition of 5 mL glacial acetic acid after cooling. The absorbance was measured at 560 nm. The contents of total saponins in WIE and WAE were calculated using Astragaloside IV as standards.

The content of Astragaloside IV in WIE and WAE was determined by a Waters Alliance HPLC system coupled with a Waters ACQUITY QDa Mass Detector (Waters Corp., Milford, USA). Samples were eluted on an Agilent Extend C-18 analytical column (150 mm × 2.1 mm I.D., 5 μm) at 25 °C with mobile phases of water-acetonitrile-formic acid (65:35:0.1, v/v/v), at a flow rate of 1.0 mL/min. Between two injections, the column was washed with 100% B for 2 min and equilibrated with the initial mobile phase for 5 min. The eluate was detected by mass spectrometry with an electrospray ion source operating in the positive ion mode (ESI^+^) using single ion recording (SIR). The monitored (M + Na)^+^ ions was m/z 807.30 for Astragaloside IV.

#### Flavonoids

Quantifications of 4 main flavonoids (calycosin-7-O-β-d-glucoside, ononin, calycosin and formononetin) in WIE and WAE were performed by a Waters ACQUITY-UPLC CLASS system (Waters Corp., Milford, USA) coupled with an ACQUITY UPLC HSS T3 column (150 mm × 2.1 mm, 1.8 µm) maintained at 30 °C. Elution was performed with a mobile phase of A (0.2% phosphoric acid in water) and B (0.2% phosphoric acid in ACN) under a gradient program: 0–1 min, 22% B; 1–10 min, 22–60% B. The flow rate was 0.35 mL/min, and the injection volume was 2 μL. The analytes were monitored at the UV wavelength of 254 nm. Between two injections, the column was washed with 100% B for 2 min and equilibrated with the initial mobile phase for 5 min.

### Animals and drug treatment

Male and female ICR mice (18–24 g) supplied by the Faculty of Healthy Science Animal Centre of University of Macau were fed on a standard laboratory diet with free access to water at a controlled temperature of 22 ± 1 °C and relative humidity of 50% with a 12 h light/dark cycle. After 1 week acclimatization, mice were randomly divided into five groups (3 male and 3 female for each group).

For experiments, the mice in Group 2 ~ 5 were received an equivalent volume of sterile PBS, WIE, WAE or UFP (twice daily, equaled to 1 g herb/kg/day) intragastrically (i.g.) for 18 consecutive days. The immunosuppression was induced by intraperitoneal injection (i.p.) of Cy (80 mg/kg/day) for three consecutive days (day-12 to day-14). In addition, mice in Group 1 (normal control group) were received and injected an equivalent volume of sterile saline with same producer for comparison. On day-19, the animals were sacrificed by CO_2_ asphyxiation, and the thymus, spleen and serum were harvested for future determinations [11]. All the experimental protocols were in accordance with the National Institutes of Health guidelines for the Care of Use of Laboratory Animals, and approved by the Animal Research Ethics Committee (reference No: UMARE-014-2017), University of Macau, Macao SAR, China.

### Body weight and peripheral white blood cells

The body weights of the experimental animals were recorded on day-1 (before drug treatment), day-12 (before Cy injection) and day-19 (before sacrifice). Moreover, the number of peripheral white blood cells were countered and recorded day-1, day-7, day-12, day-14 and day-19.

### Immune organ index

The thymus and spleen were immediately excised surgically and weighed. The immune organ index was calculated according to the following formula:

$${\text{Thymus or spleen index }}\left( {{\text{mg}}/{\text{g}}} \right)\, = \,\frac{{{\text{Weight of thymus or spleen }}\left( {\text{mg}} \right)}}{{{\text{Body weight }}\left( {\text{g}} \right)}}$$

### Preparation of splenocytes

Splenocytes were obtained by gentle disruption of the mice spleen and filtration with a 100-μm Nylon cell strainer (Falcon, NJ, USA) [12]. The erythrocytes were lysed with ACK lysis buffer, and the splenocytes were collected by centrifugation 400×*g* at 4 °C for 5 min (Thermo Fisher, MA, USA). After washing three times with PBS, the splenocytes were re-suspended in RPMI 1640 complete medium with 10% fetal bovine serum, 100 μg/mL streptomycin, and 100 IU/mL penicillin. The viability of the cells was determined to be higher than 95% using the trypan blue dye exclusion technique.

### Splenocyte proliferations

Splenocyte proliferation was determined using CCK-8 Cell Counting Kit. Splenocytes (5 × 10^5^ cells/well, 100 μL) in serum free RPMI-1640 medium were seeded onto a 96-well plate. The cells were incubated in the absence of or in the presence of concanavalin A (ConA, 5 μg/mL) or lipopolysaccharide (LPS, 10 μg/mL) for 72 h at 37 °C in a humidified atmosphere of 5% CO_2_. Thereafter, the cells were further incubated at 37 °C for 1 h after addition of 10 μL of CCK-8 solution, and the absorbance was measured at 450 nm with the reference at 650 nm.

### Natural killer (NK) cell activity

Natural killer cell activity assay was performed according to the method as previously described [12] with minor modifications. Briefly, splenocytes effector (2 × 10^6^ cells/well) were cultured with YAC-1 target (Cell Bank of Chinese Academy of Science, Shanghai, China) (4 × 10^4^ cells/well) (Target:Effector of 1:50), blank medium (normal control) or 1% NP-40 (maximum release) in a 96-well plate for 4 h at 37 °C in a humidified atmosphere of 5% CO_2_. After centrifuged at 400×*g* for 5 min, the activity of LDH in supernatant was determined using the LDH Cytotoxicity Detection Kit (ThermoFisher Scientific Inc., USA) according to the manufacturer’s instructions. NK cell activity (%) was calculated with the detected absorbance at 490 nm using the following equation:

$${\text{NK cell activity }}\left( \% \right)\, = \,\frac{{{\text{Abs}}_{\text{sample}} - {\text{Abs}}_{\text{control}} }}{{{\text{Abs}}_{{{\text{NP}}40}} - {\text{Abs}}_{\text{control}} }} \cdot 100\%$$

### Flow cytometric analysis for splenic lymphocyte subset

Cell staining for phenotypic analysis was carried out as described previously [13]. In brief, splenocyte suspension (1 × 10^6^ cells/mL) was blocked by mouse serum for 15 min and incubated with 3 μL FITC-conjugated rat anti-mouse CD3 mAbs, APC-conjugated anti-mouse CD4 mAbs and PerCP/Cy5.5-conjugated anti-mouse CD8a mAbs for 20 min at 4 °C. Splenocytes were collected by centrifugation at 300×*g* for 5 min and were washed with PBS. Splenocytes resuspended with 200 μL PBS were immediately undergone flow cytometric analysis. The percentage of positively stained cells, determined over 10,000 events, was analyzed by a FACScanto flow cytometry system (BD, New Jersey, USA). Splenocytes were identified by their characteristic appearance on a dot plot of FSC versus SSC and electronically gated to exclude platelets, red cells or dead cell debris. The results are shown as the percentage of positive cells within a gate.

### Serum IgG and IgM assays

The levels of IgG and IgM in serum were determined using the immunoturbidimetry following the manufacturer’s instruction. Briefly, the collected mice sera were diluted with phosphate buffer added PEG6000 for incubation at 37 °C for 5 min. The change of absorbance at 340 nm and 600 nm before and after incubation were measured for calculating the contents of IgG and IgM, respectively.

### Statistical analysis

Each experiment was performed in triplicate and was repeated for at least thrice. All results were presented as mean ± SD. The statistical significance of data was determined using one-way ANOVA and *t* test. A *p *< 0.05 was considered statistically significant difference.

## Results

### Whole ingredients extract shows higher extraction efficiency than water extract

The extraction efficiency and main ingredients for WIE and WAE were determined and summarized in Table 1. Although WIE (31.27%) presented a slightly higher extraction efficiency than that of WAE (30.43%), the extraction of total polysaccharides, total saponins, and individual ingredients (astragaloside IV, calycosin-7-O-β-d-glucoside, ononin, calycosin and formononetin) in WIE were all higher than those in WAE, suggesting the complete extraction of such ingredients using gradient solvent extraction.

### Whole ingredients extract reverses Cy-induced decrease of body weight and organ index in immunosuppressive mice

**Fig. 1:**
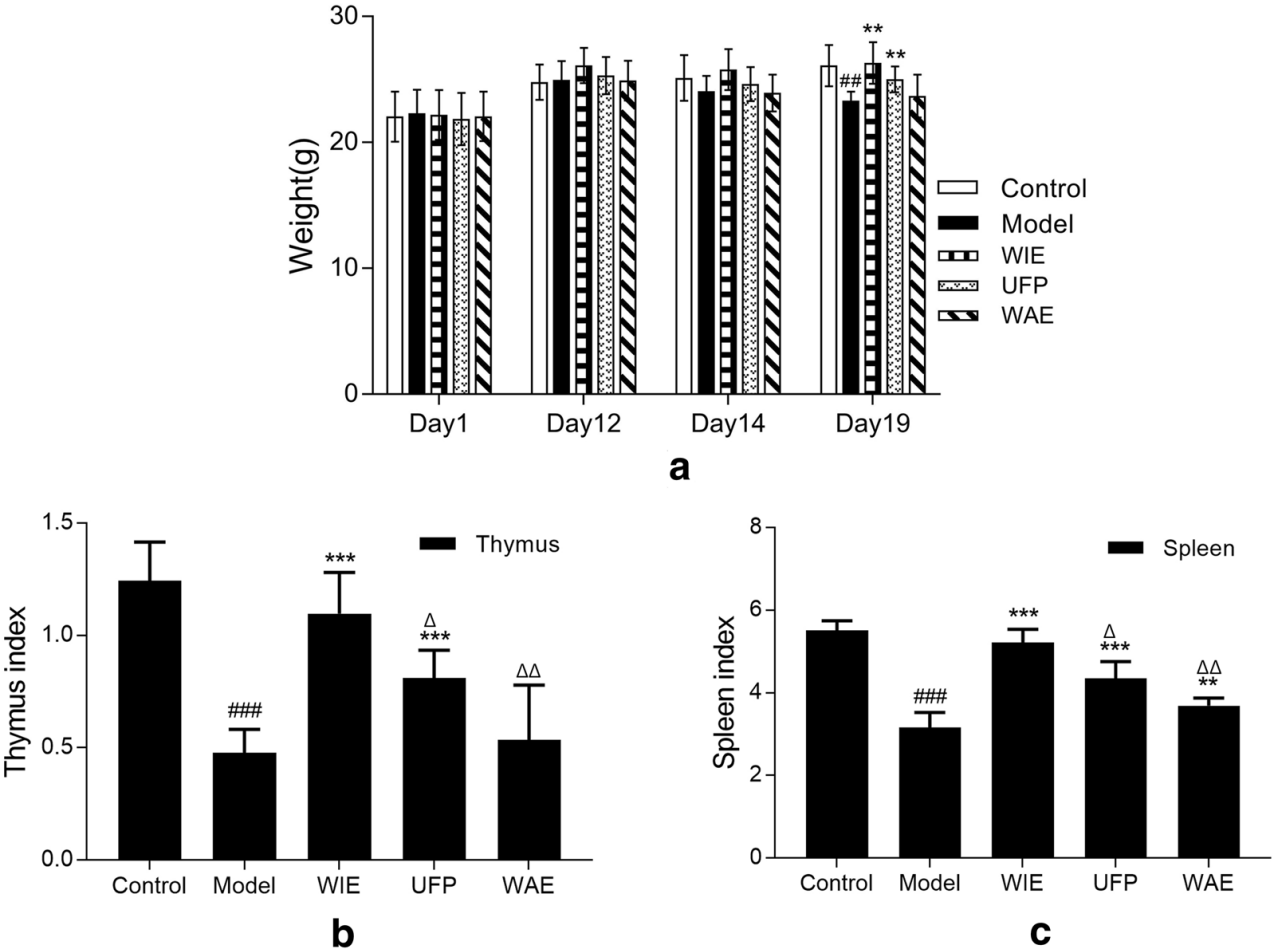
**a** Body weights of mice before and after intragastric administration with physiological saline, whole ingredients extract (WIE), water extract (WAE) or ultrafine powder (UFP) (twice daily, equalled to 1 g herb/kg/day) for 18 days with cyclophosphamide (Cy, 80 mg/kg/day) injected intraperitoneally from days 12–14. **b** Thymus index and; **c** Spleen index for different groups of mice. Data are expressed as mean ± S.D. of six mice. ^##^*p* < 0.01 vs Control. *p < 0.05, **p < 0.01 and ***p < 0.001 vs Cy; ^Δ^*p* < 0.05 and ^ΔΔ^*p* < 0.01 vs WIE

The average body weight of each group on day-1, day-12, day-14 and day-19 were illustrated in Fig. 1a. In the first 12 days and the completed day of Cy injection, no significant difference on growth was observed for all animals. After immunosuppression with Cy (day-14 to day-19), the body weight of mice were quickly reduced. However, the reduction of body weight in immunosuppressive mice could be antagonized by treatment of Astragalus preparations, and with the efficacy of WIE ≥ UPF > WAE.

In addition, the relative weight of immune organs in Astragalus-treated groups were determined to be significantly improved while compared to those in the immunosuppressive group. As shown in Fig. 1b, c, the highest potency on increasing thymus and spleen indexes was observed for WIE, and followed by UFP and WAE.

### Whole ingredients extract increases number of peripheral white blood cell and serum levels of immunoglobulins

**Fig. 2:**
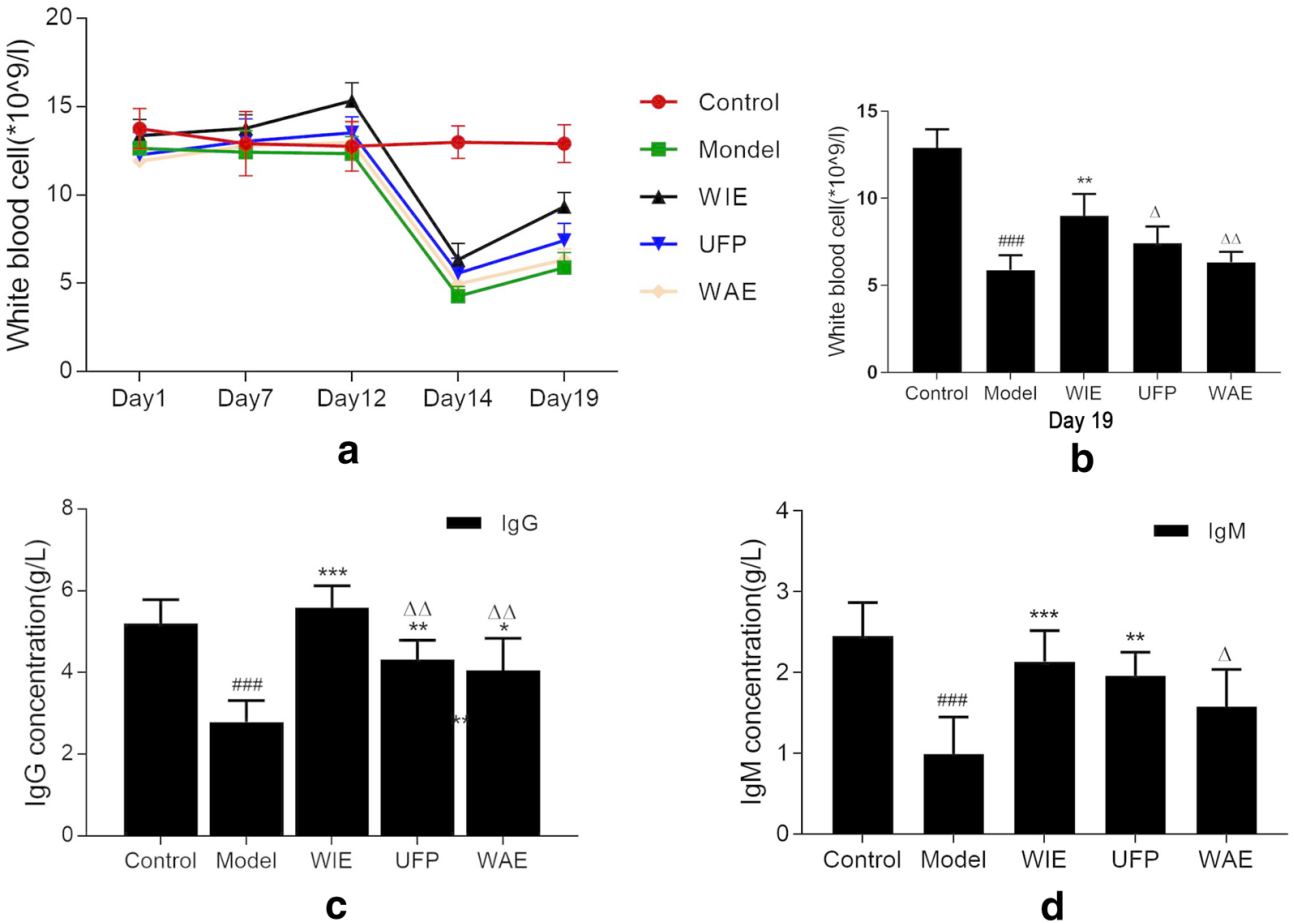
**a** Timely changes of peripheral white blood cell (PWBC, × 10^9^/L) during experimental process: intragastric administration with physiological saline, whole ingredients extract (WIE), water extract (WAE) or ultrafine powder (UFP) (twice daily, equalled to 1 g herb/kg/day) for 18 days with cyclophosphamide (Cy, 80 mg/kg/day) injected intraperitoneally from days 12 to 14. **b** PWBC level at Day 19. **c** Serum IgG and **d** serum IgM levels in different groups of mice. Data are expressed as mean ± S.D. of six mice. ^###^*p* < 0.001 vs Control; *p < 0.05, **p < 0.01 and ***p < 0.001 vs Cy; ^Δ^*p* < 0.05 and ^ΔΔ^*p* < 0.01 vs WIE

As shown in Fig. 2, the number of peripheral white blood cell (PWBC) (Fig. 2a, b) and serum levels of immunoglobulins (IgG and IgM) (Fig. 2c, d) in mice were determined to be markedly reduced after immunosuppressing by i.p. injection of Cy. However, oral administration with Astragalus preparations could effectively enhance both the PWBC numbers and the serum immunoglobulins levels in immunosuppressive mice. The efficacy of WIE was comparable to or better than those of UFP, and much better than those of WAE.

### Whole ingredients extract improves splenic T and B lymphocyte proliferations and cytotoxic activity of natural killer cells

**Fig. 3:**
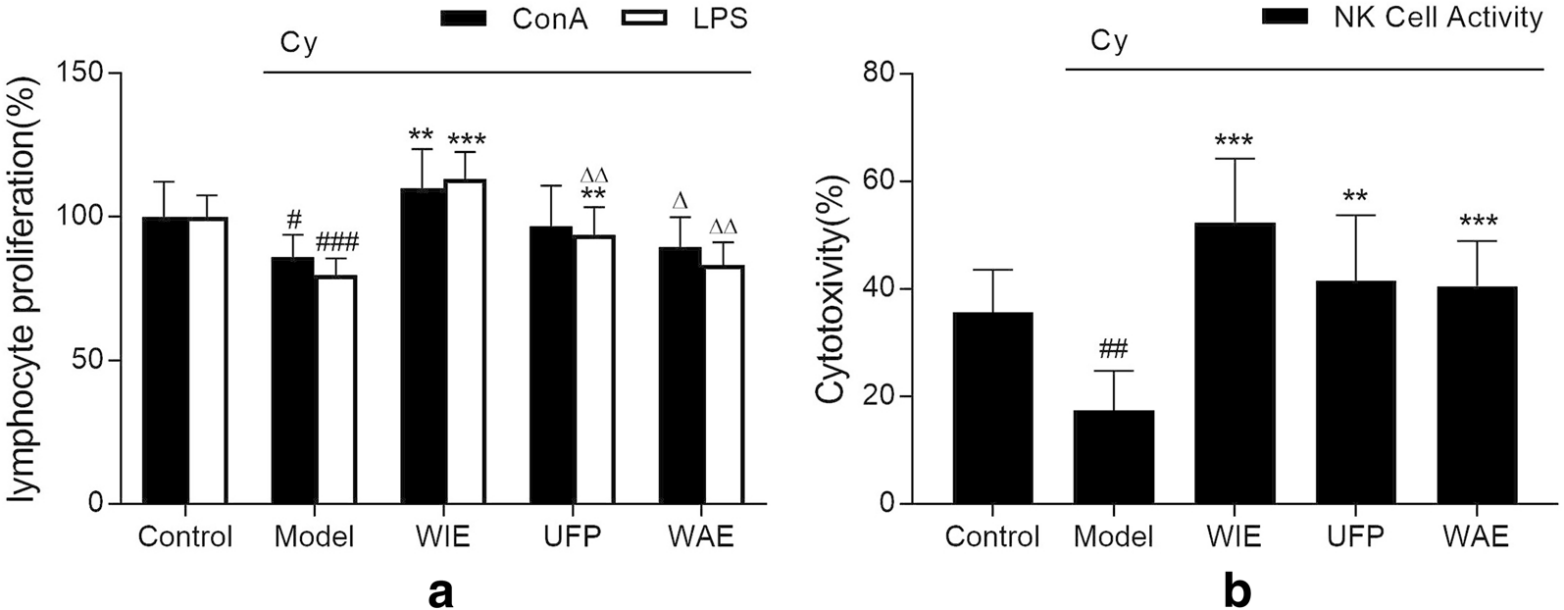
**a** Concanavalin A (ConA, 5 μg/mL) or lipopolysaccharide (LPS, 10 μg/mL)-induced splenic lymphocyte proliferation in mice having intragastric administration with physiological saline, whole ingredients extract (WIE), water extract (WAE) or ultrafine powder (UFP) (twice daily, equalled to 1 g herb/kg/day) for 18 days with cyclophosphamide (Cy, 80 mg/kg/day) injected intraperitoneally from days 12 to 14. **b** Natural killer cell (NK cell) activity of splenocytes in different groups of mice. Data are expressed as mean ± S.D. of six mice. ^#^*p* < 0.05, ^##^*p* < 0.01 and ^###^*p* < 0.001 vs Control, **p < 0.01 and ***p < 0.001 vs Cy; ^Δ^*p* < 0.05 and ^ΔΔ^*p* < 0.01 vs WIE

Both T and B lymphocyte proliferations triggered by concanavalin (ConA) and lipopolysaccharide (LPS) were augmented by WIE in Cy-induced immunosuppressive mice (Fig. 3a). UFP improved B lymphocyte proliferation but not T lymphocyte proliferation. Besides, WAE had no significant effects on proliferation of both lymphocytes. In addition, the cytotoxic activity of splenocytes against natural killer (NK)-sensitive YAC-1 cells was lowered in Cy-treated mice, and was promoted by all three types of Astragalus preparations comparable to normal control mice (Fig. 3b).

### Whole ingredients extract increases both splenic CD3^+^ and CD4^+^ T lymphocytes

**Fig. 4:**
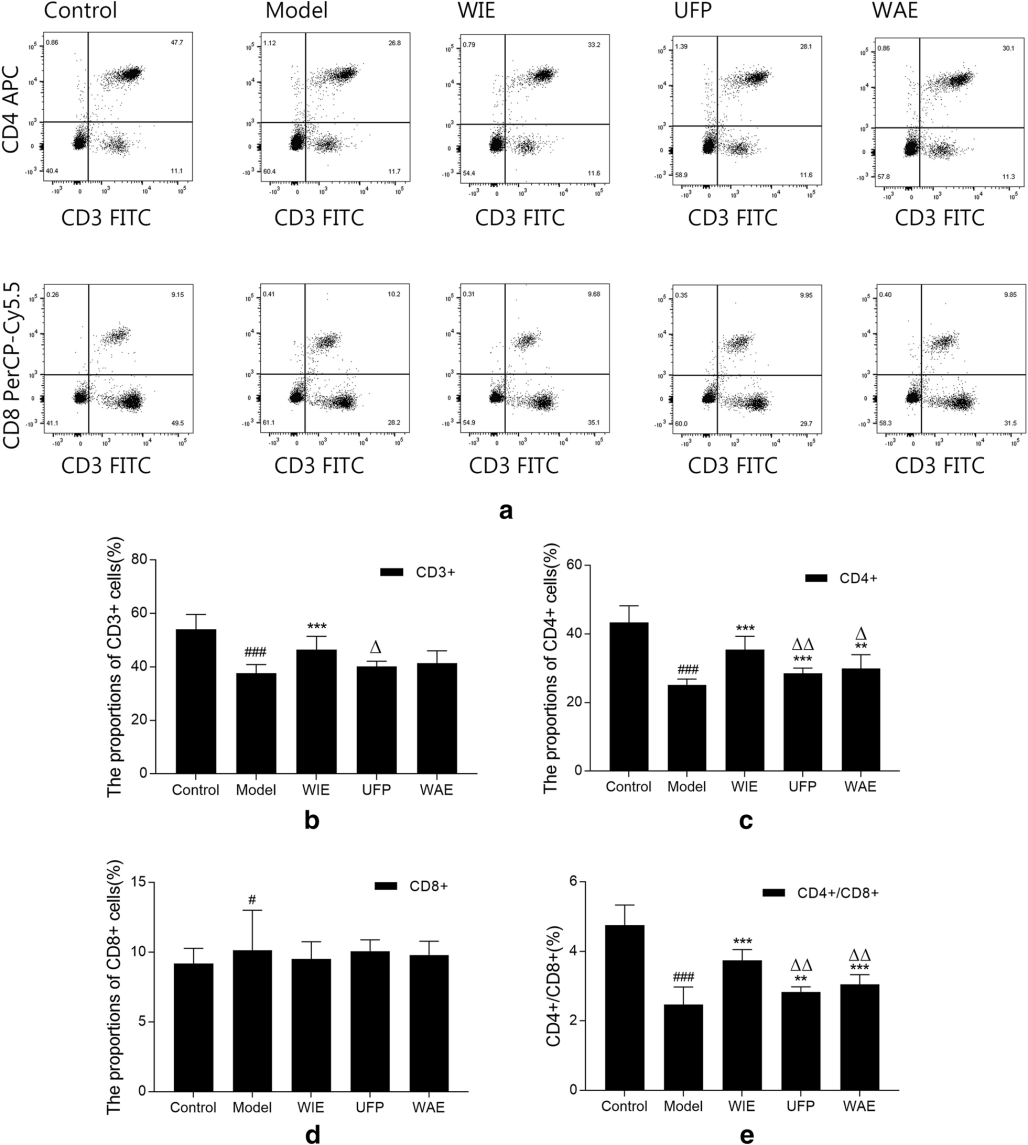
**a** Representative images of splenic T lymphocyte counts determined by flow cytometry for mice having intragastric administration with physiological saline, whole ingredients extract (WIE), water extract (WAE) or ultrafine powder (UFP) (twice daily, equalled to 1 g herb/kg/day) for 18 days with cyclophosphamide (Cy, 80 mg/kg/day) injected intraperitoneally from days 12 to 14. Summarized graphs for (**b**) proportion of CD3 + , **c** proportion of CD4 + , **d** proportion of CD8 + , and (E) the ratio of CD4 +/CD8 + . Data are expressed as mean ± S.D. of six mice. ^#^*p* < 0.05, and ^###^*p* < 0.001 vs Control; **p < 0.01 and ***p < 0.001 vs Cy; ^Δ^*p* < 0.05 and ^ΔΔ^*p* < 0.01 vs WIE

Immunophenotype of splenocytes were evaluated by counting CD3^+^, CD4^+^ and CD8^+^ T lymphocytes using flow cytometry (Fig. 4a). The percentages of CD3^+^ and CD4^+^ lymphocytes, and the ratio of CD4^+^/CD8^+^ were determined to be significantly decreased in Cy-injected mice (Fig. 4b–e). Treatment with WIE reversed the percentages of CD3^+^ and CD4^+^ lymphocytes and CD4^+^/CD8^+^ ratio. Furthermore, UFP and WAE increased splenic CD4^+^ lymphocytes but had no effect on CD3 + lymphocytes. There was no obvious difference among groups for CD8^+^ lymphocytes (Fig. 4d).

## Discussion

In the past decades, plant-derived products have been more and more popular and acceptable for medical treatment and health maintenance. The medicinal/dietary plants, especially some traditional Chinese herbal medicines, are highly concerned because of their satisfied functions and good safety which have been demonstrated by long-term clinical applications. However, the quality and efficacy of such products are unevenness due to the process and quality of the related herbal extract. Therefore, development of high quality raw materials (i.e. herbal extract) is important and necessary.

Traditionally, water and a certain concentration of organic solvents (e.g. ethanol) are the most commonly used solvents for preparing of herbal extracts. Due to the wide-range difference in physicochemical properties of the active ingredients containing in the herbs, part of which are always lost during the extracting process due to their poor solubility in the solvent. For example, high contents of water-soluble ingredients (polar macromolecules and small molecules) but low contents of alcohol-soluble ingredients (weak polar and non-polar small molecules) are often determined in the water extract (WAE) of herbs. The disintegrity in chemistry of the extract suggested the loss of certain pharmacological functions while compare to those of the herb itself.

In recent years, ultrafine powder (UFP) of herbal material has been quickly developed and applied as new raw materials for healthcare products. Under a low temperature, the herbal materials are grinded into a small particles (D90 < 45 µm), which reached to a cytoderm level broken. The chemical integrity of the UFP could be well retained, and presented a high correlation coefficient to that of the herbal decoction pieces [5]. Moreover, the bioavailabilities of the active compounds in UFP were investigated to be higher than those in normal herbal powders because of their quick and completed dissolution from the UFP [6, 7]. The advantages of UFP allows a lower dose unit in a single dosage thus improving the compliance in clinical applications. However, the some potential problems in safety for UFP, such as the irritations of the powder to gastrointestinal mucosa etc., are more and more concerned in recent years. Therefore, development of a new pharmaceutical materials by combining the advantages of WAE and UFP might be another strategy for improving the clinical applications of Chinese medicines.

Astragalus is a traditional tonic herbal medicine and has been widely investigated both in chemistry and pharmacology [14, 15]. The immunomodulatory effects of Astragalus are demonstrated to be associated with various chemicals including polysaccharides [16], saponins [17] and flavonoids [18]. In the present study, we prepared a whole ingredients extract of Astragalus (WIE) by using the gradient solvent extraction approach. Compare to the traditional WAE, WIE improves both the total extraction efficacy and the integrity of the chemical composition. Furthermore, the efficacy of WIE, WAE and UFP in modulation of immunity was determined using Cy-induced immunosuppressive mice model. The dosage for drug administration was set at 1 g herb/kg/day, which is equalled to 40 percentage of the recommended median dosage in Chinese Pharmacopoeia on human (15 g/person/day). Of note, the actual amounts administrated for WIE and WAE were 0.31 g/kg/day and 0.30 g/kg/day, respectively, due to the extraction process (extraction yield of 31.27% and 30.43%) while the amount was retained for UFP at 1.00 g/kg/day. The dose unit at the same dosage of both extracts were lower than that of UFP.

Cyclosporine (Cy) is a cytotoxic chemotherapeutic drug for tumor therapy to cause myelosuppression and immunosuppression, and lead to anemia resulting from erythro-poietin deficiencies [19]. Toxicities of Cy include the suppression of white blood cells, nausea, vomiting, gonadal atrophy, liver, renal, and bladder injury [20]. Our preliminary experiments showed that i.p. injection of 40 or 150 mg/kg/day Cy for three days caused too slight or irreversible change of PWBC while 80 mg/kg/day was chosen as a proper dosage for immunodeficiency model, of which the PWBC level decreased significantly but almost recovered to its normal status 5 days after the last injection. In this study, WIE showed better effects to increase PWBC count and spleen and thymus indexes than UFP or WAE, suggesting its protection against immunosuppression and leukopenia. Notably, WIE was able to increase PWBC in mice not only after Cy injection but also before Cy injection as compared with those administered with saline. The results implied that chronic consumption of WIE of Astragalus can prevent or retard the development of immunosuppresion and can reduce the damage caused by a sudden decrease in immune function.

IgG and IgM are two major immunoglobulins to humoral immune responses and play important roles in complement activation, opsonization, and neutralization of toxins [21]. Long term oral administration of WIE, UFP or WAE augmented the serum IgG and IgM levels in immunosuppressive mice. The present results are consistent with a previous report describing that Astragalus polysaccharides prevented against the immunosuppression by upregulating the secretion IgG and IgM [22].

Another widely used parameter for immune system is to measure T and B lymphocyte proliferation induced by two mitogens, ConA and LPS respectively, due to its high sensitivity [23]. Our preliminary experiments supported the dose-dependent stimulatory effect of ConA and LPS for lymphocyte proliferation and 5 μg/mL of ConA and 10 μg/mL of LPS were chosen as effective concentration (data not shown).

NK cells are large, granular lymphocytes derived from bone marrow and reside primarily in the spleen, capable of recognizing and clearing foreign, infected, damaged, or malignant cells through various of mechanisms [24, 25]. NK cell activity is an important index for non-specific immune system which has a close relation to anti-tumor and anti-virus infection, and also participates in allergic reactions and immunity [26]. NK is regulated by T, B lymphocytes and bone marrow stem cells, and releases cytokines such as interferon (IFN)-γ and tumor necrosis factor (TNF)-α to modulate natural and specific immune responses [27]. All WIE, UFP and WAE promoted NK cell activity but WIE was the most effective one, revealing the amelioration of the host immune system for both adaptive and innate immunity.

T cells consist of T helper (Th) cells, T cytotoxic (Tc) cells, T suppressor cells, and T effector cells. CD4^+^ and CD8^+^ T cells are markers of Th and Tc lymphocytes involved in adaptive immunity [28]. The proportions of CD3^+^, CD4^+^ and CD8^+^ T cells were observed and the ratio of CD4^+^/CD8^+^ was calculated, illustrating that WIE, UFP and WAE modulated T cell functions whereas WIE showed the best regulatory effect.

In this study, Cy injection reduced the body weight, spleen and thymus indexes, PWBC, serum levels of IgG and IgM, splenocyte proliferation, NK cell activity, and splenic lymphocyte subsets. All the results indicated that the immunosuppressive mice were successfully introduced and are consistent with previously studies [29, 30]. We demonstrated that 18-day i.g. administration of WIE, UFP or WAE all accelerated the recovery of immunosuppressed mice from leukopenia and immunosuppression, supported by (1) the increased PWBC and spleen and thymus indexes, (2) normalized serum immunoglobulin level, (3) augmented splenocyte proliferation and NK cell activity, and (4) enhanced splenic CD3^+^ and CD4^+^ T lymphocyte subsets. Importantly, the potency of such immunomodulatory were observed to be WIE ≥ UFP > WAE.

## Conclusions

A new WIE of Astragalus was developed and comprehensively investigated both in chemistry and pharmacology. WIE has better chemical integrity than WAE; and at the same time, presents more comparable or better potency to restore impaired immunity in mice at the same dosage than those of UFP and WAE. The obtained results suggested that WIE combines the chemical and pharmacological characteristics of WAE and UFP, and presents more advantages in safety, effectiveness, stability, controllability as well as compliance than those of the traditional water extract and ultra-fine powder. This reveals that WIE has great potentials to be further developed as promising and novel raw material or food supplement for pharmaceutical products and functional foods.

## Additional file

**Additional file 1.** Minimum standards of reporting checklist.

## Abbreviations

WAE: water extract
UFP: ultrafine powder
WIE: whole ingredients extract
Cy: cyclophosphamid
RPMI-1640: roswell park memorial institute 1640
LPS: lipopolysaccharide
ConA: concanavalin A
CCK-8: enhanced cell counting Kit-8
IgG: immunoglobulin G
IgM: immunoglobulin M
HPLC: high performance liquid chromatography
ESI^+^: positive ion mode
SIR: single ion recording
UPLC: ultraperformance liquid chromatography
ACN: acetonitrile
PWBC: peripheral white blood cell
PBS: phosphate-buffered saline
NK Cell: natural killer cell
LDH: lactate dehydrogenase
SD: standard deviation
ANOVA: analysis of variance
