# Correction to: Immunomodulatory effects of a new whole ingredients extract from Astragalus: a combined evaluation on chemistry and pharmacology

**DOI:** 10.1186/s13020-021-00440-3

**Published:** 2021-05-06

**Authors:** Zhi Xin Li, Guan Ding Zhao, Wei Xiong, Ke Gang Linghu, Qiu Shuo Ma, Wai San Cheang, Hua Yu, Yitao Wang

**Affiliations:** 1 Institute of Chinese Medical Sciences, State Key Laboratory of Quality Research in Chinese Medicine, University of Macau, Macao, China; 2 HKBU Shenzhen Research Center, Shenzhen, Guangdong China; 3 School of Chinese Medicine, Hong Kong Baptist University, Kowloon Tong, Hong Kong, China; 4 Institute of Chinese Medical Sciences, University of Macau, Room 8008, Building N22, Avenida da Universidade, Taipa, Macao SAR China; 5 Institute of Chinese Medical Sciences, University of Macau, Room 1050, Building N22, Avenida da Universidade, Taipa, Macao SAR China

## Correction to: Chin Med (2019) 14:12 10.1186/s13020-019-0234-0

Following the publication of the original article [1], it was noted that Table 1 is missing.

**Table 1:** Extraction amounts (g/100 g herb) of main compounds in different Astragalus extracts

Extract	Total polysaccharides (g/100 g herb)	Saponin (g/100 g herb)		Flavonoid (g/100 g herb)			
		Total	Astra IV	F I	F II	F IIII	F IV
WIE	12.87 ± 0.032	0.52 ± 0.022	0.011 ± 0.001	0.010 ± 0.002	0.003 ± 0.001	0.039 ± 0.001	0.013 ± 0.001
WAE	11.52 ± 0.027	0.25 ± 0.019	0.012 ± 0.001	0.004 ± 0.001	0.001 ± 0.001	0.019 ± 0.003	0.004 ± 0.001

*Astra IV* Astragaloside IV, *FI* calycosin-7-O-β-d-glucoside, *FII* ononin; *FIII* calycosin; and *FIV* formononetin

*WIE* whole ingredients extract, *WAE* water extract

The table and caption have been included in this correction, and the original article has been corrected.
